# 

**Published:** 1903-02-15

**Authors:** 


### ANNOUNCEMENTS.

### THE OHIO DENTAL SOCIETY.

THE officers elected by the Ohio Dental Society at its last meeting were: *President*—Dr. J. B. Beauman, Columbus. *First Vice-President*—Dr. J. T. Stephan, Cleveland. *Second Vice-President*—Dr. W. T. McLean, Cincinnati. *Secretary*—Dr. S. D. Ruggles, Portsmouth. *Treasurer*—Dr. C. I. Kelly, Hamilton.

### THE ODONTOLOGICAL SOCIETY OF WESTERN PENNSYLVANIA.

The Odontological Society of Western Pennsylvania will meet in Pittsburg, Tuesday and Wednesday, March 10th and 11th. The Executive Committee is endeavoring to induce several of the best men in the profession to contribute to the program. The exhibits will be numerous and interesting. The largest attendance in the history of the society is promised for this meeting. C. B. Brath, Pres.

### DRS. W. H. WHITSLAR AND GEO. H. WILSON.

Drs. W. H. Whitslar and Geo. H. Wilson, of Cleveland, have moved into the large new office building at the corner of Euclid Av., and Erie streets, "The Schofield."

